# Lewis Carroll's Doublets Net of English Words: Network Heterogeneity in a Complex System

**DOI:** 10.1371/journal.pone.0114177

**Published:** 2014-12-17

**Authors:** Hsieh Fushing, Chen Chen, Yin-Chen Hsieh, Patrick Farrell

**Affiliations:** 1 Department of Statistics, University of California Davis, Davis, California, United States of America; 2 Amsterdam University College, Amsterdam, Netherlands; 3 Department of Linguistics, University of California Davis, Davis, California, United States of America; UMIT, Austria

## Abstract

Lewis Carroll's English word game Doublets is represented as a system of networks with each node being an English word and each connectivity edge confirming that its two ending words are equal in letter length, but different by exactly one letter. We show that this system, which we call the Doublets net, constitutes a complex body of linguistic knowledge concerning English word structure that has computable multiscale features. Distributed morphological, phonological and orthographic constraints and the language's local redundancy are seen at the node level. Phonological communities are seen at the network level. And a balancing act between the language's global efficiency and redundancy is seen at the system level. We develop a new measure of intrinsic node-to-node distance and a computational algorithm, called community geometry, which reveal the implicit multiscale structure within binary networks. Because the Doublets net is a modular complex cognitive system, the community geometry and computable multi-scale structural information may provide a foundation for understanding computational learning in many systems whose network structure has yet to be fully analyzed.

## Introduction

From an evolutionary perspective, language may be the most interesting evolutionary trait of the human species and of human cultures [1]. Aided by advances in informational technology, recent research efforts have greatly expanded the reach of the scientific study of language, as well as our overall understanding of languages, cultures, and the human psyche as complex, structured systems. Just as in such “hard” sciences as mathematics, statistics, and physics, access to big data with sophisticated computational tools has allowed social scientists in disciplines such as linguistics, anthropology, and psychology to both answer old questions and open new doors of inquiry that were not even imaginable two decades ago.

One of the things about language that makes it amenable to computational analysis is that its components are hierarchically organized, with smaller units being combined into more complex units whose organization reflects the application of rules at several different levels [1]. The most basic levels in the grammar of a language are phonology, which is concerned with how words and their component pieces may be composed from units of sound (or phonemes), morphology, which has to do with how words are built out of the more elementary units known as morphemes (or the roots, prefixes, and suffixes contained within words), and syntax, which has to do with how sentences are built out of words. The rules and constraints governing these levels of grammar have embedded in them multiple layers of informational content embodying vast amounts of semantic, communicative, cultural, logical, and computational knowledge.

For the quantificational analysis of language and culture, the recent book digitization project at Google Inc. constitutes a monumental step forward. This project provides an enormous collection of written language texts that is publicly accessible via the Google Books Ngram Viewer (https://books.google.com/ngrams/info), which provides analyses of word and phrase frequency over time. A 1-gram is a string of characters uninterrupted by a space (roughly, a word) taken from over 15 million books. An *n*-gram (roughly, a phrase or sentence) is a sequence of 1-grams of size *n*, with *n* being unbounded. As such, Google Books is “big data” par excellence that provides a solid foundation for the new field of culturomics, or the study of human behavioral and cultural trends based on digitized texts, and gives us numerous ways of examining human evolution as it is encoded in the stories and legends of numerous languages and their corresponding cultures. Linguists, anthropologists, and psychologists are looking increasingly to statistics and computational linguistics for answers to vexing questions of longstanding. And, with the assistance of sophisticated mathematical models, high-throughput data collections such as Google Books are certain to enable them to approach satisfying answers to many such questions. Indeed, the Ngram Viewer has already made it possible for researchers to enhance their analyses of different cultures and languages with a variety of quantificational techniques. A recent analysis of the Google Books database [2] yielded revealing case studies of individual words that provide new avenues for pursuing issues in cultural anthropology, as fluctuations in the meanings and frequencies of words are essentially waypoints in the map of a culture. This influential work was quickly followed by several very interesting works that used the same database with different focuses, including an analysis of the aggregate properties of word-birth and word-death dynamics based on a yearly word-count time series on a logarithmic scale [3], a macroscopic analysis of the decreasing need for new words based on the same word-count data format [4], and an analysis of how the popularity of the most common English words and phrases evolves [5].

These quantitative analyses are all primarily concerned with the evolution of word frequency along the temporal axis. This paper adds a new dimension, as it focuses on one global structural pattern of English morphology and phonology seen through the lens of a relative microcosm of data taken from the fourth edition of the American Heritage Dictionary of the English Language, and focusing only on the spellings of words, as listed in this database, as they relate to a word game. Although this corpus of English word spellings is but a slice of the language, it is also clearly a complex dynamic system that reflects selection forces that have been operative in the evolution of the language dating to circa 500 CE, and even before that, given that they are partially conditioned by systematic features of the Germanic and Indo-European ancestors of English and universal constraints on the structure of human languages. Thus, even though the corpus is a cross-sectional snapshot of English words, the structural rules extracted via the data-driven computations proposed here arguably reflect and reveal inherent patterns in the selection forces that have brought one part of the language to where it currently is as a system, as well as where it will go in the future.

The computational technique employed here is network-based. Since the key ingredient in a recipe for understanding networks is the inter-relational connectivity among all its nodes, a computational analysis of the structural relationships between the spellings of English words in the corpus examined allows key aspects of the morphological and phonological systems that condition them to become visible. Naturally, using a different corpus and a different measure of relational connectivity to construct a network for the language is bound to yield a somewhat different global view. For instance, Perc [6] studies the evolution of meaning relationships between common English word and phases using the Ngram Viewer and shows that the Matthew effect yields a global view that is very different from what is discussed here. Moreover, culturomics rooted in network-based computational linguistics will surely offer perspectives on the evolution of human cultures that are likely to be quite different from those based primarily on word frequency (see [7]). Nevertheless, it is to be hoped that the collective coupling of global views from cultural, semantic, syntactic, morphological, and phonological perspectives will help us to more fully understand the structure of the entire network of English language and culture from the computational standpoint.

## Results and Discussion

Charles Lutwidge Dodgson, better known by the pseudonym Lewis Carroll and his 1865 book *Alice's Adventures in Wonderland*, created an English word game on Christmas Day in 1877 [8]. This game, which can currently be found on several websites under different names, including Doublets, Word Ladder and Word-Links, is played by first choosing two words, and then going in steps from one to the other by changing only one letter and creating a legitimate English word at each step. For instance, for the word pair “LOVE” and “HATE”, one simple successful path has two intermediate steps: “HOVE” and “HAVE”. Some pairs of words take many more steps, such as “ROGUE” and “BEAST”, for which Lewis Carroll provides the following solution, with ten intermediate steps: “ROGUE” [VOGUE, VAGUE, VALUE, VALVE, HALVE, HELVE, HEAVE, LEAVE, LEASE, LEAST] “BEAST”. There also exist extremely difficult pairs, such as “INCA” and “ADIT” (a kind of entrance to an underground mine), which needs at least 19 steps, as well as word pairs for which there are no solutions.

The way to systematically solve Doublets word puzzles is to use network analysis. To illustrate, we took all the 4-letter English words from a word list and constructed a network that can be represented by a symmetric binary adjacency matrix. Each entry is equal to “1” if the pair of words is connected, otherwise to “0”. By wandering through edges from node to node in such a network, via a series of basic matrix operations, we can provide solutions for all potential word pairs in the game and can determine the degree of difficulty for each solution. Specifically, for any pair of nodes it is possible to specify the length of the shortest path or paths and this path length indicates the difficulty of this pair. Roy-Floyd-Warshall's algorithm [9], [10] can then be used to generate a matrix of such path lengths for all possible node pairs, such that the degree of difficulty can be easily identified prior to playing the game.

This network is of interest not so much because it provides solutions to the puzzles in this game, but because it displays certain distinctive features that have not yet been well studied in network-related research on social, mathematical, biological and physical systems. Three features of particular interest are:

**1**. No two nodes are identical.

**2**. Each edge is deterministic and functionally distinct.

**3**. Each node's degree is far below its capacity.

The last two of these appear to be characteristic of linguistic knowledge more generally, as they appear in semantic networks for modeling relationships between word meanings [10]–[12] as well as word-form networks for modeling the phonological and orthographic patterns in lexicons ([13]–[15]).

**Figure 1:**
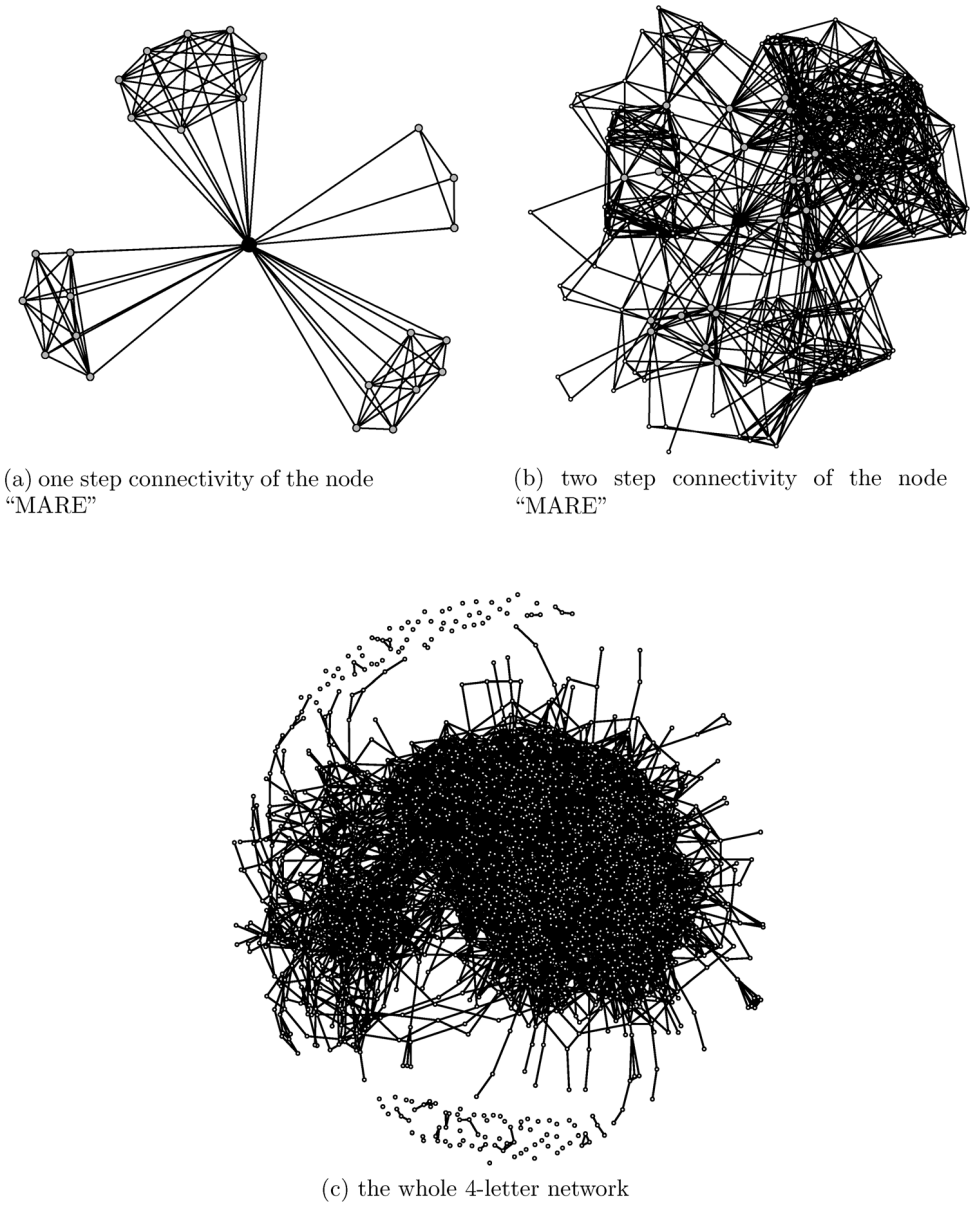
Local to global views of the 4-letter word network. Typical patterns of network connectivity from the view of a node to its immediate neighbors (a), and the view to its two step neighbors (b), and the overall view of the whole network (c).

To better see these features and their implications, we can zoom in on the network of all 4-letter words and look at the connectivity of one node, say “MARE”, as shown in Fig. 1(a). There are 25 edges, in all, connecting to the node “MARE”, for which reason we say that the degree of this node is 25. The 25 immediate connections from “MARE” are divided exactly into four small groups that are each defined by one of the four letter slots in the word. That is, there are 8 out of 25 ( = 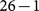) possible choices of English letters that can substitute for “M” in the first slot of “?-A-R-E”. Likewise, there are 3 for “M-?-R-E”, 7 for “M-A-?-E” and 7 for “M-A-R-?”. This kind of limitation on letter combinations illustrates the third distinctive feature of the system, which is especially important. The word “MARE” in fact has the largest degree among all 4-letter words. Yet, it only reaches one fifth of its capacity of 100 ( = 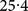). Hence, this network is unlikely to be equipped with the so-called scale-free degree distribution, which would prescribe a power law of 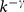 for its heavy tail indexed by degree 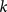 and a positive constant 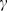 (see [16], [17]). On the other hand, 75 out of the 100 possible single letter changes are impermissible due to linguistic constraints that increase redundancy or efficiency [18], [15]. For instance, “M-L-R-E” violates a letter-combinatoric (orthographic) constraint specific to English that reflects a set of sound-combinatoric (phonological) constraints that is only partially specific to English. The bilabial nasal consonant phoneme, for which the International Phonetic Alphabet symbol is/m/and which is usually indicated by the letter “M” in the English spelling system, can only be followed, when word-initial, by a vowel phoneme. There is, in effect, a systematic phonological and orthographic conspiracy against not only “M-L-R-E”, but also “M-T-R-E”, “M-S-R-E”, “M-N-R-E”, and so forth. The main components of this conspiracy are: an English-specific constraint with the effect of forcing the nasal phoneme/m/to be a consonantal part of the onset of a syllable when it is word-initial; universal constraints on consonant-vowel (C-V) structure that disallow most syllable onsets of the form C-C-C-, including/m-C-r-/[19], [20]; and an English-specific orthographic constraint limiting the set of single-character representations of vowel phonemes to the letters “A”, “E”, “I”, “O”, “U” and “Y”, even though the language actually has upwards of thirteen vowel phonemes in all, with the precise number depending on the dialect and exactly how the count is made.

Another kind of systematic constraining force on the degree of nodes in the network can be seen mostly in larger words with more than one syllable and more than one morpheme. Consider, for example, the 6-letter word “ACTION”, which consists of two syllables and two morphemes: the root “ACT” and the suffix “-ION”. In this case, the spelling is not a very good representation of the sound structure, since there is no/t/sound in the word and the letter “I” does not represent a vowel sound. For historical reasons, having to do with its Latinate origin, “ION” just happens to be the spelling of a noun-forming suffix that attaches to the verb “ACT” and many other verbs, and typically changes the pronunciation of the root-ending consonant. Most importantly, because of the particular morphological structure of this word, all three of the letters following “T”, if “ACT” itself is not changed, must represent a legitimate morpheme (or sequence of morphemes) that can occur as suffixes on the verb “ACT”. Thus, given “A-C-T-?-?-?”, the only possible words with the last three slots filled are “ACTION”, “ACTING”, “ACTORS”, “ACTANT”, “ACTIVE” and “ACTUAL”. Since none of these words has an “N” in the last slot, there is no possible substitution for the “O” in “A-C-T-I-O-N”, even though there is no phonological or orthographic constraint against “ACTIAN” or “ACTIEN”, for example. Limits on the number and kind of prefixes and suffixes in the language, on how they are conventionally spelled [21] and on their combinatorial power play a major role, alongside phonological constraints, in structuring the writing system and are responsible for a lot of the degree weakness of nodes in the Doublets net.

While “M-T-R-E” and “M-N-R-E” violate phonological constraints, given English spelling conventions, and “A-C-T-I-L-Y”, for example, violates a morphological constraint preventing the suffix combination spelled “ILY”, as in “HANDILY”, from attaching to verbs, there are also many apparently accidental gaps in the English word inventory. “MURE”, for example could be a word, pronounced/mjur/, with the diphthong/ju/in the vowel slot, by analogy with “MUSE” and “PURE”. However, it simply has not been conventionalized as the spelling of a word, although the word “MUIR”, with the same pronunciation, has. Similarly, “MYRE”, pronounced/majr/, with another diphthong in the vowel slot, could be a word, by analogy with “LYRE”/lajr/, but it is not, although “MIRE”, with the same pronunciation is. Since the abundance of available word spellings greatly exceeds both the number of distinct pronunciations of actual words in the language and the need for more words, many potential spellings of words naturally go unused.

It appears that linguistic constraints of the kind noted above, which have the effect of balancing efficiency and redundancy, are parts of a generic selection force that has been shaping the English language for centuries. This selection force helps us to comprehend the distinctions among the 25 edges linking to the node “MARE” and the reduction of 75 in its degree capacity. It also fundamentally differentiates the network analysis considered here from most studies of complex systems in the recent network literature.

When focusing on any word connecting to “MARE”, the selection force mounts to work out its local connectivity in a heterogeneous and complex fashion. The two-step connectivity of the node “MARE” offers a glimpse into such heterogeneity and complexity, as shown in Fig. 1(b). One area is much more densely connected than the others. This area is formed by merging the two subnetworks developed based on the 8 nodes resulting from changes to the first consonant letter and the 7 nodes resulting from changes to the second consonant letter. This contrasts with the much less dense subnetwork based on the 3 vowel changes. Taking into account all 4-letter words, we have the network shown in Fig. 1(c). This network has a rather uncommon characteristic. There is a giant connected clique with many attached dendrites and a small portion of nodes that are either isolated or have few connections. Such a giant clique is also found in phonological networks for English words [22]. Here a connected clique means that any two of its nodes have at least one path leading from one to the other via existing edges. It is noted that, by simply choosing two ending nodes from two separate dendrites attaching to this giant clique, as with “ADIT” and “INCA”, mentioned above, it is possible for a pair of 4-letter words to yield only rather long paths. This kind of pair would surely lead to much head scratching and frustration for Doublets players. In any case, the interesting question that arises is how a dendrite comes to exist. We propose a selection-force answer to this question below.

The density of links within communities in contrast to the sparseness of between-community links, coupled with the the complicating presence of dendrites, yields a kind of heterogeneity that seems to obscure the heuristic validity of the notion of community that has been in use for decades in social network research. In sharp contrast to all community detection techniques in the literature up to now, here we take a general computational approach to developing an intrinsic network-driven node-to-node “distance” measure that might be used for any binary network under study. Based on such a distance concept, we further develop a computational algorithm, called community geometry, to bring out the multiscale structure that is otherwise implicitly embedded within the binary network. This community geometry allows us to answer the question of whether community A is closer to community B than it is to community C. It also resolves the problem of overlapping community membership to a great degree. While we focus here on questions of word structure and their relationships to computed communities, we anticipate that this intrinsic node-to-node distance concept and the community geometry developments could facilitate a better understanding of other complex systems, for which more than a game puzzle is at issue.

Going further, this community geometry implies that the question of how many communities are contained in a binary network is actually ill-posed, because a multiscale community structure is one that has a different community configuration when seen from different focal scales in the network, in much the same way that the structure seen in an organism varies with the resolving power of a microscope. Different focal scales show different structural information. However this simple fact seems not to have been considered properly in the network literature. Even in the recent discussion of the importance of multiscale structure [23], only multiple known and explicit physical scales are recognized.

Detailed comparisons of our community geometry with results derived from modularity, a popular community detection methodology [24]-[26], would take us too far afield here and is the focus of a separate report in preparation. Briefly, however, although modularity is capable of distinguishing densely connected subnetworks from less densely connected areas by iteratively optimizing an ad hoc target function, in our opinion, it does not and cannot bring out so-called authentic community structure [27], [28] with geometric perspective. We also note that a community construct found via a context-free similarity measure defined between links (see [29], [30]) is not suitable for a setting like the Doublets net, in which nodes and functions of links are all diverse and distinct.

When focusing on the whole system, the Doublets net exhibits a spectacular evolving structure overall. Constructed from a database of nearly 60,000 English words, it is a series of networks derived from words with letter length ranging from 3 to 22. How the selection force works in this system can be seen by contrasting word pairs that differ greatly by number of syllables but only minimally by number of letters. For example, the one-syllable word pair “DIVE” and “WIPE” is linked via a 2-step path: “DIRE”, “WIRE”. The two-syllable pair “DIVER” and “WIPER”, on the other hand, is connected by the following 6-step path: [RIVER, RAVER, CAVER, CAPER, PAPER, PIPER]. This example illustrates an overall pattern whereby path length tends to correlate with the number of linguistic units contained in the word pairs, including the number of syllables. From an information-theory perspective, this pattern is the result of two mutually counteracting forces in the evolution of vocabulary: efficiency and redundancy. Attaching additional syllables to existing words is clearly a way to increase the vocabulary capacity of a language. At the same time, however, it introduces heavier selection forces, especially due to redundancy. We employ this evolutionary perspective to argue that the Doublets net is a system in which a nearly single giant clique evolves into several less dense cliques, then transforms into a complete scattering of disconnected single words, and ends abruptly with the complete absence of words that are too long.

In the Conclusion section, we consider some implications of recognizing the Doublets net as a modular complex system. Since linguistic knowledge of English word structure is contained in the Doublets net in a distributed fashion and this knowledge can only be retrieved by computing from various resolutions, our multiscale computing is a model of computational learning in a complex system. We anticipate that this kind of computational learning model will provide a foundation for exploring many real complex systems whose precise content and structure are not fully known and the computational techniques developed here may therefore be useful for exploring many other aspects of knowledge.

## Analysis

In this section we use classic network analysis to explain the three distinctive features of the Doublets net and illustrate their significance with the giant clique of the 4-letter word network shown in Fig. 1(c). Classic network components, such as path length, clustering coefficient, betweenness and degree distribution, are all valid and useful in the Doublets net. But their usefulness is especially salient with respect to its three distinctive features. We also comment, in passing, on why this network is characteristically different from a small-world network and a network with scale-free degree distribution.

### Classic and adapted network analysis

**Figure 2:**
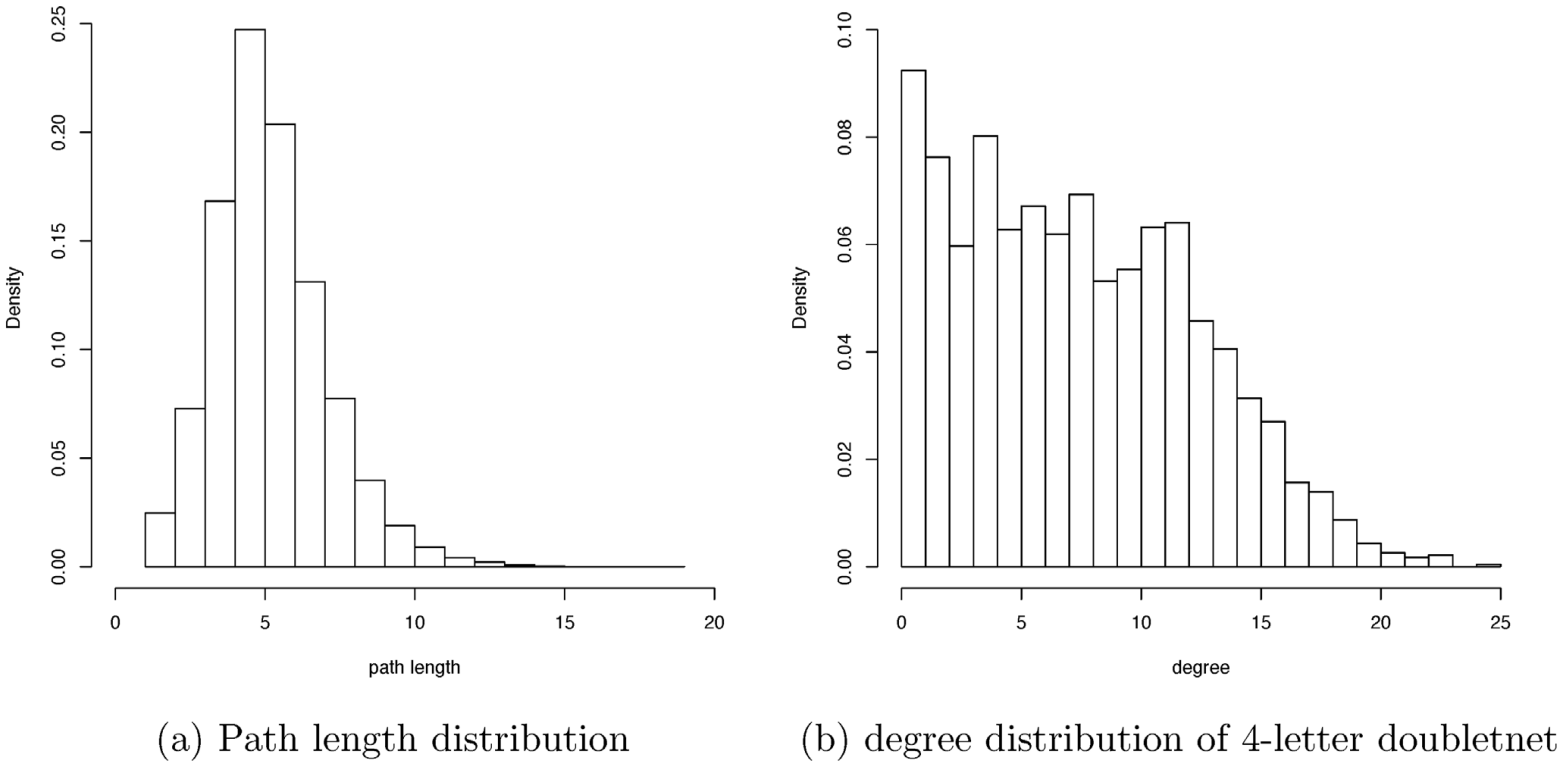
Path length and degree distributions. Histogram of path length of the 4-letter giant clique given in Fig. 1 (c) (a) and its histogram of degree (b).

The average path length of the 4-letter word giant clique is calculated as around 5. So, in general, this game is not particularly difficult with a 4-letter word setting. But, as shown in Fig. 2(a), Doublets can be extremely difficult for some 4-letter words. The most difficult ones are those pairs involving ending nodes of dendrites, such as the aforementioned “ADIT” and “INCA” pair. Here we attempt to answer the question of how a dendrite is formed, in order to shed light on some special characteristics of the Doublets net.

A dendrite consists of a series of low degree nodes. The ending node has degree one, while intermediate nodes have degree two. As shown in the 2 o 26 contingency table in Table 1, 4-letter words starting with a vowel, which we call V-words, are more likely to have a small degree (between 0 and 3) than a word starting with a consonant, which we call a C-word. And, a 4-letter V-word is more likely to have two syllables than a C-word. Thus, the selection force seems to exert heavier pressure on V-words than on C-words. Under this pressure, a V-word is more likely to link to low degree C- or V-words with similar sound combinations, and then the same pattern seems to repeat. Therefore, we postulate that the chain effect of having a heavier selection force on V-words and low degree C-words is the primary driving mechanism for dendrite formation.

**Table 1:** Degree vs. word type for 4-letter words.

Degree													
	0	1	2	3	4	5	6	7	8	9	10	11	12
C-word	41	70	112	111	151	131	147	129	153	119	126	141	147
V-word	52	49	63	26	33	13	7	13	6	3	1	4	0
	13	14	15	16	17	18	19	20	21	22	23	24	25
C-word	104	93	71	62	36	32	20	10	6	4	5	0	1
V-word	1	0	1	0	0	0	0	0	0	0	0	0	0

The presence of many dendrites then makes the Doublets net unlike a small-world network. Also, as shown in Fig. 1(a), all the nearest neighbors of any node would be separated into four distinct and unconnected “clusters”, which is not a small-world characteristic, since it makes the clustering coefficient rather heterogeneous. Further we see that the degree distribution in Fig. 2(b) has a rather short tail. This phenomenon is primarily due to the third distinctive feature.

Consider next the possibility of betweenness being centered around an edge. Betweenness of one edge is defined as the proportion of shortest paths among the whole collection containing this particular edge. The giant clique of 4-letter words has much more than 2 million 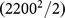 shortest paths, while only having around 9000 edges. A high betweenness edge acts like a bottleneck tunneling from one densely connected subnetwork on one side to another densely connected subnetwork on the other side. What kind of edge is more likely to have high betweenness? Important clues to this question are revealed in the 3 o 3 contingency table, shown in Table 2.

**Table 2:** High betweenness and edge type.

	vc-edge	vv-edge	cc-edge	total
VC edge	19(9.170)	0(0)	0(0)	19(9.170)
VV edge	19(2.854)	8(2.247)	27(7.634)	54(12.735)
CC edge	186(47.116)	82(48.239)	111(342.374)	379(430.095)
TOTAL	224(59.140)	90(50.486)	138(342.374)	452(452)

Prevalence of functionally different edges in the top 5% in terms of high betweenness, compared to random baseline numbers (in parentheses).

Let's call one edge with two C-words on both ends a CC-edge, and use the same convention to define VV- and VC-edges. In contrast, the edge being formed by changing a consonant letter into another consonant letter is called a cc-edge, yielding a similar definition for vv- and vc-edges. We distinguish the subpopulation of edges within the top 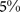 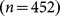 of high betweenness by using 9 functionally different edge categories in the contingency table. Accordingly, for the baseline comparison shown in the parentheses, we randomly select an equal number of edges and make the same classification. Table 2 clearly shows that the vc-edges are most closely related to high betweenness, with the vv-edges ranked second, followed by VC- and VV-edges.

The implication of these results, taken together, is that vowel letters have been subject to a more significant and rather distinct selection force than consonant letters have. Since each high betweenness edge renders a less dense connectivity region, when a collection of such regions forms continuous belts, they naturally divide the whole network into communities. It is also interesting to note that, due to the heterogeneity in connectivity density, some communities mount to be closer than others. This is the geometric foundation of community with multiscale structure within a single network. A computation algorithm that actually computes this community geometry is developed in the next subsection.

### Community geometry

Even in recent network literature, a community residing in a network is still intuitively perceived as a group of nodes that are more densely connected in contrast to their “surrounding regions” where connectivity is relatively sparse. Due to the lack of a quantitative measurement to ascertain the degree of density or sparseness in connectivity, there exists no rigorous definition of community. Nonetheless, a network has its given and deterministic structure. If a community structure indeed exists and is embedded within a network under study, wouldn't this structure also be given and determined by the network as a whole? In this section, we attempt to answer this question affirmatively.

**Figure 3:**
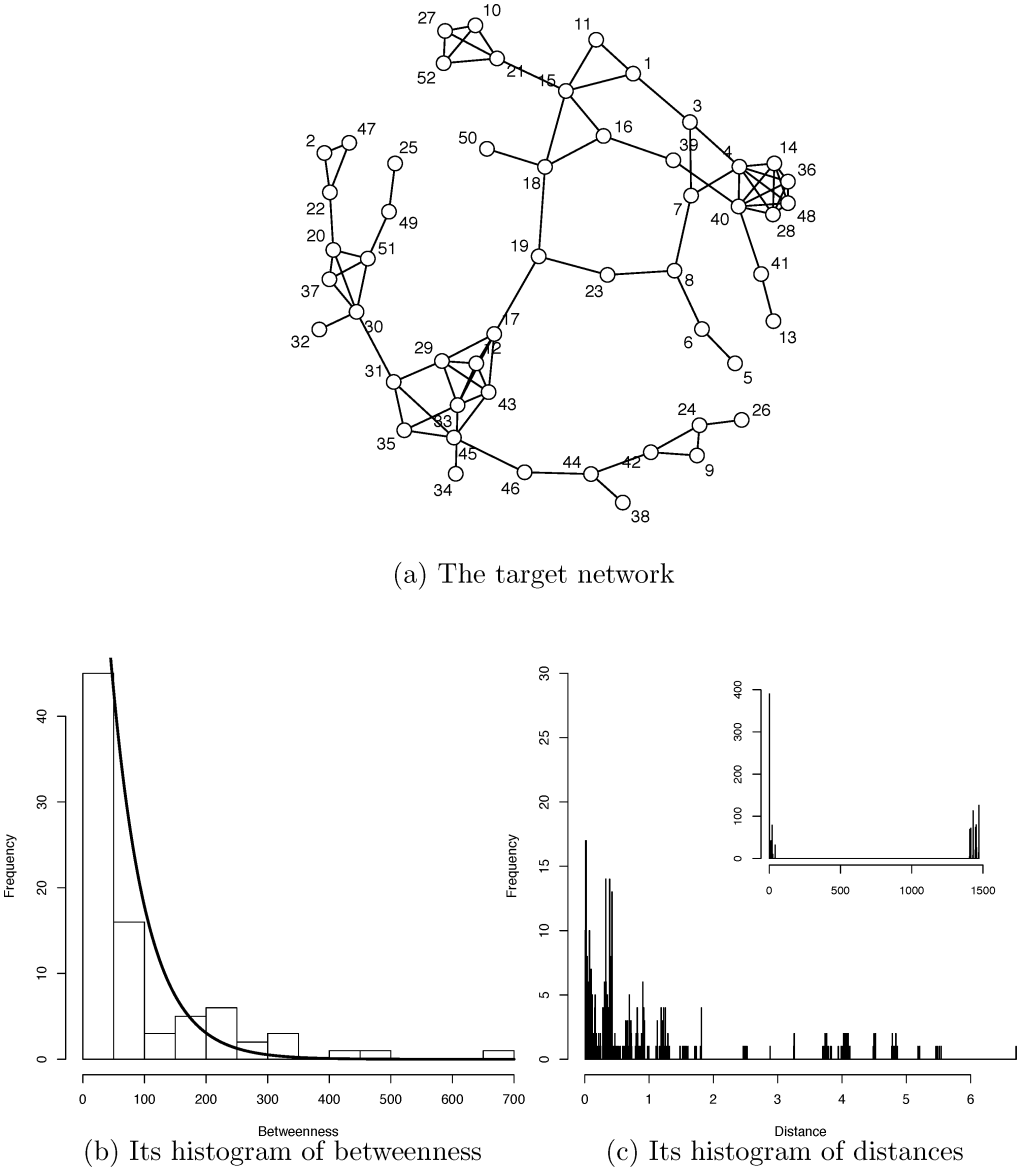
A network and its betweenness and node-to-node distance. The community geometry of the second largest clique in the 8-letter network (a) is computed from its histograms of edge betweenness (b) and node-to-node distances (c).

We first develop a natural and explicit measure of “distance” among all nodes, given a binary network. This distance measure not only facilitates the discovery of communities, but also induces another, implicit “distance” concept that allows us to determine whether community A is closer to community B than to community C. Thus a final computational result for a binary network is a community geometry. It then becomes clear that this community geometry reflects and mirrors many ad hoc and artificial facets of results from popular community detection methodologies, such as modularity. Furthermore, the community geometry also brings out the multiscale structural information in a network. To our knowledge, this is the first such attempt of its kind in network research. Our methodological development is illustrated here with a simple network, as shown in Fig. 3(a), which is the second largest clique of 8-letter words in our Doublets net. All of its word nodes are numbered and given in Table 3.

**Table 3:** Words in the second largest component in the 8-letter network.

1	2	3	4	5	6	7	8
babbling	boggling	bubbling	bumbling	bundling	bungling	burbling	burgling
	10	11	12	13	14	15	16
cackling	cobbling	dabbling	dangling	dumpling	fumbling	gabbling	gambling
	18	19	20	21	22	23	24
gangling	garbling	Gargling	giggling	gobbling	goggling	gurgling	hackling
	26	27	28	29	30	31	32
haggling	heckling	hobbling	humbling	jangling	jiggling	jingling	juggling
	34	35	36	37	38	39	40
mangling	mantling	mingling	mumbling	niggling	pickling	rambling	rumbling
	42	43	44	45	46	47	48
rumpling	tackling	tangling	tickling	tingling	tinkling	toggling	tumbling
	50	51	52				
waggling	warbling	wiggling	wobbling				

**Table 4:** Algorithm 1.

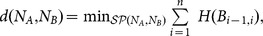

#### Betweenness-based node-to-node distance

A node-to-node distance concept is developed in three steps. First, consider the highest betweenness edge on the network in Fig. 3(a), which is found at the edge linking node 17 as “gangling (17)”, and node 19 as “gargling (19)”. It is obvious that there is a community on both sides of this edge, since no connectivity could be found within the surrounding region separating the two groups of nodes, except this “bridge” edge. We also empirically see other high betweenness edges, such as “jingling (31)” to “jiggling (30)” and “tingling (45)” to “tinkling (46)”. Connected to these two edges we also observe three smaller communities that make up one of the two communities separated by the highest betweenness edge. Intuitively these three communities should be closer to each other than they are to the community on the other side with the node “gargling”. A high betweenness edge reveals community formation information and, at the same time, different values of betweenness also give information about degree of closeness as well. The idea of using betweenness for community detection was first employed by Newman [27]. Later this idea was abandoned, presumably due to an unsettled issue concerning number, in favor of the idea of modularity and an eigenvector of 0-1 adjacency matrix approach [25]. The question of closeness between communities has never been pursued.

Second, from the illustrating network we see that the two connected nodes “gangling” and “gargling” must have a large distance in order to be separated and belong to two different communities. This distance should be larger than that of the pair “jingling” and “jiggling” and the pair “tingling” and “tinkling”, because the betweenness of the latter two is smaller. This leads us to conclude that distance must be an increasing function of betweenness. Third, any non-directly connected pair of nodes would render at least one shortest path going from one to the other. Consider the pair “dabbling (11)” and “mingling (35)”. Apparently, their shortest path indeed includes the “bridge” edge of “gangling” and “gargling”. Hence the distance between these two nodes must be larger than the distance between the nodes of “gangling” and “gargling”, since they are further apart. On the other hand, consider the different pair “dabbling” and “wobbling (52)”. These two nodes are likely in the same community and their shortest path does not involve the bridge edge. Hence, their distance should in turn be much smaller that that of “gangling” and “gargling”.

By synthesizing the three aspects discussed above, we formally propose a measure of node-to-node distance as follows. Let 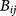 denote the betweenness of a directly connected pair of nodes 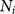 and 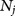. Consider two target nodes, 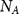 and 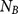, with path length n and one of their shortest paths identified as 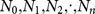 with 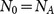 and 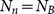. We define the distance between 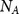 and 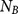 in Algorthm 1 in Table 4, where 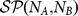 denotes the collection of shortest paths between 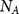 and 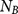, and the kernel function 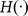 would be empirically constructed in the following way.

Conceptually this kernel function 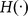 has to make sure that small, but positive 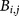 is mapped into a nearly zero value, while a relatively large betweenness will be mapped to a large value. In order to express this intuition that there is compressing of betweenness values on one side, but stretching on the other side, we collect all available betweenness values of all directly connected pairs and construct a histogram, as shown in Fig. 3(b). We fit an exponentially decreasing function to capture the tail behavior on the right and a nearly vertical asymptote on the left, as shown by the solid line in Fig. 3(b). The reciprocal of this fitted exponential tail function is used as the kernel function 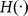. For this illustrative example, the computed kernel function is 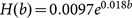. The distance 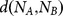 defined through this simple data driven kernel function turns out to work very well for the purpose of constructing community geometry.

#### Algorithm for constructing community geometry

With 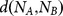 defined for a binary network, its community geometry can be constructed with a computational algorithm called data cloud geometry, developed by Fushing and McAssey [31] for clustering purposes. In a data cloud geometry, a set of temperature scale values (*T*'s) are identified when the geometry undergoes phase transitions. Here the phase transition of concern is community merging. Hence, a realized “distance” between two communities can be inferred to be equal to the particular scale value *T* when the two communities indeed merge together. Therefore, the ordering according to size of communities merging as *T* is the essence of the multiscale structure pertaining to the community geometry under study. A brief synopsis of the process for computing community geometry, and its motivations, are given below.

Given a temperature *T*, a measurement of similarity between nodes 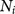 and 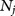 is defined by Algorithm 2 in Table 5. From this definition, we see that nodes 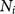 and 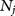 would have a larger similarity value, i.e., 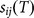 comes closer to 1, to the extent that 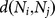 is smaller than *T*. The higher the similarity value, the more likely the two nodes are to be in the same community, under the scale value *T*. By contrast, to the extent that 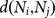 is larger than *T*, 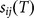 is smaller and the two nodes are more likely to be in different communities. From this perspective, by varying the scale *T*, we are able to explore the multiscale structural of the original binary network. So, the next question is how to empirically find a set of potential and relevant scale values for *T*.

**Table 5:** Algorithm 2.

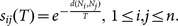

An answer to this question is found in the information residing on the tail of the histogram of pairwise node-to-node distances. As shown in Fig. 3(c), there is a series of bumps located along the tail. And the bump locations are good candidates for *T* scales. The underlying reason is that when two nodes are in the same community, the distance between them is short. This aggregation gives rise to a large bump near 0. Beyond this baseline bump, each bump residing on the tail is an aggregation of node-to-node distances potentially belonging to two communities. Thus, the formation of bumps is largely due to several communities being heterogeneously located. This heterogeneity, in turn, suggests that multiscale structure among communities should be considered. We confirm this in the next subsection, where multiscale community structures for two subnetworks of the Doublets net are examined.

The community structure pertaining to a scale *T* can be computed as follows. With the computed similarity 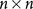 matrix 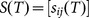, the original binary network 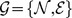 is transformed into a weighted complete graph 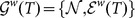 with 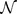 standing for all involving nodes, 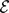 for all observed binary edges and in contrast 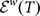 for all 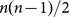 edges weighted by 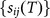
's. Upon 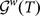 a Markovian transition probability matrix is derived as 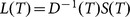 with diagonal degree matrix 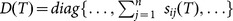. 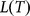 is also called a normalized Lapacian in the literature on clustering. The algorithmic computations for the data cloud geometry (DCG) can be summarized briefly as follows.

**DCG-1**: First, a regulated random walk is built based on the Markovian transition matrix *L*(*T*) to explore the community membership information among all involved nodes. The key idea is that a *L*(*T*)-based random walk at any given time point is more likely to stay within a community than to jump to another community. While traveling within a community, the regulated random walk is designed to remove nodes one-by-one, whenever a node has accumulated visits beyond a fixed threshold. This removal eventually exhausts one community, and then forces the random walk to jump to another community. This regulated random walk continues removing nodes until nearly all have been removed. The recording of such a stochastic removal process creates a profile of the recurrence time of node removals. A high spike in this profile indicates that the regulated random walk has just entered a new community. Hence, nodes that are removed between two spikes are likely to be in the same community. This is a piece of essential membership information for community detection under a fixed scale *T*.

**DCG-2**: By repeating the regulated random walk exploration numerous times, we can collectively estimate the probability of two nodes being in the same community. This matrix of empirical probability estimates is called an ensemble connectivity matrix under scale *T*. We then compute the eigenvalue plot of this ensemble connectivity matrix to extract information concerning the number of communities involved. Also, we create a hierarchical clustering tree (with complete modularity) based on this ensemble connectivity matrix, in order to accurately characterize the community membership.

**DCG-3**: By increasing the scale values of *T* on the series of empirically selected bump locations, we get a process of core-to-conglomerate merging. At the lowest *T* value, there might be many core communities. As *T* is increased, we see that core communities begin to merge into conglomerate communities. A core or conglomerate community that is closest to another community at a scale *T* merges first as *T* goes up to the next larger value. The recording of such merging processes yields a community structure hierarchy. This hierarchy of multiscale community structures is called a community geometry.

The next subsection turns to an illustration of how a community geometry is constructed, with real data analysis, for two networks.

#### Computed community geometry

We first compute the community geometry of the illustrative example network, shown in Fig. 1(a). To do this, we compute the betweenness for all nodes and the shortest path and node-to-node distance for all pairs. Also we choose a set of potential scale values for *T* as 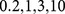 based on the tail behavior shown in Fig. 3(c). Then we apply the data cloud geometry algorithm.

**Figure 4:**
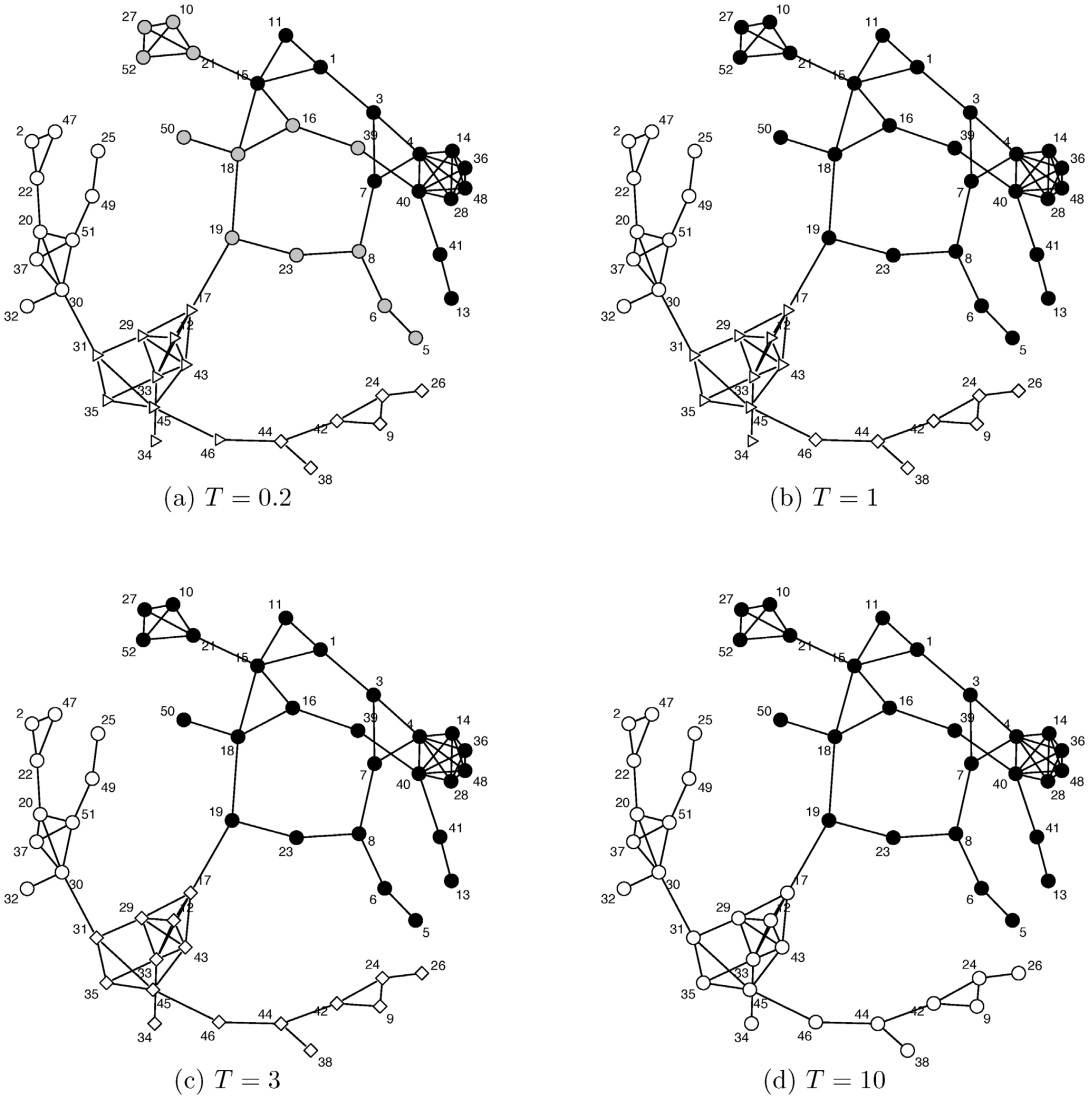
The community geometry of the second largest clique in the 8-letter network. Four 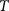 scale values are used to bring out the community geometry of the second largest clique in the 8-letter network. The merging process starts from 5 core communities, and then 4, 3 and 2 on the four panels.

At 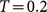, we find five core communities as shown in Fig. 4(a). As expected the five communities result from cutting the high betweenness edges, including the three mentioned earlier. At 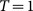, two core communities are merged into a conglomerate one. The resulting four communities are shown in Fig. 4(b). This merging indicates that these two core communities are the closest among the five core communities at scale 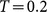. Hence the distance between the two core communities could be loosely said to be equal to 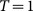. As we raise the scale value of *T*, the communities continue merging and finally, at 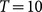, the edge of the node pair “jingling” and “jiggling” is recovered to form a conglomerate community consisting of three core communities.

The structure of the final two conglomerate communities is a consequence of the cut on the edge of the node pair “gangling” and “gargling”, shown in Fig. 4(d). It is important to note that these two communities reveal a phonological pattern: all but one of the members of the community that includes the node “gangling” have a consonant letter immediately following the vowel that represents a velar phoneme, i.e., a sound that is produced by raising the tongue body to the back part of the roof of the mouth (the velum), as in the case of “giggling”/giglıŋ/, where the double “gg” represents a single velar sound, or as in the case of “gangling”/gæŋglıŋ/, where the letter “n” represents the velar nasal consonant sound/ŋ/, which is forced to be velar by the following velar/g/sound, due to a quasi-universal phonological process known as nasal assimilation. By contrast, all but one of the members of the other community have a consonant letter representing something other than a velar sound immediately following the first vowel. With the one exception aside, they either have the letter “r”, representing an approximant sound produced with the tongue raised forward of the velum; the letter “m” followed by “b” or “p”, representing a cluster of bilabial sounds, i.e., sounds with the two lips touching; or a pair of “b” letters representing a single bilabial consonant sound, as with “bubbling”.

These patterns confirm that the community geometry indeed brings out relatedness in terms of phonological structure. Phonological constraints limit the clustering of consonant sounds, following principles that maximize harmony between neighbors, and this is reflected in spelling regularities, albeit in less than completely transparent ways, given that “n” can represent either a velar or an alveolar sound. But, it is precisely because of this idiosyncrasy of English spelling that there are some exceptional nodes in the communities. On phonological grounds, the node “mantling”/mæntlıŋ/“belongs” in the non-velar community, but isn't there, and “bungling”/b
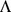
ŋglıŋ/“belongs” in the velar community, but isn't there. This is due to the ambivalent nature of the letter “n”, which undermines a purely phonological definition for these particular communities and shows why knowledge of spelling conventions and their complex relationship to pronunciations is embodied alongside phonological knowledge in the Doublets net.

**Figure 5:**
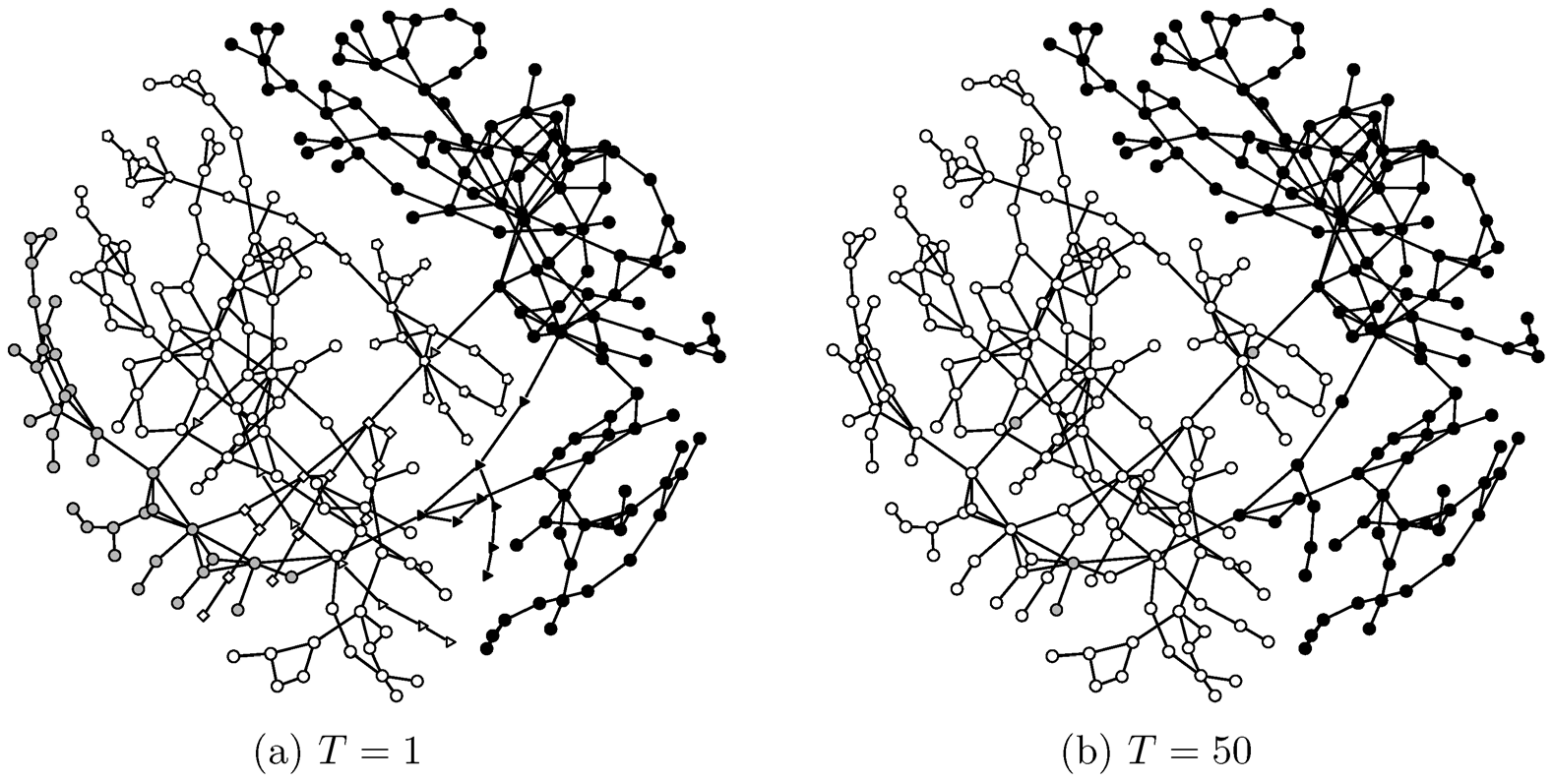
The community geometry of the largest clique in the 8-letter network. The community geometry of the largest clique in the 8-letter network is shown for only two 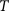 scale values. The merging process starts from 8 core communities on the left panel and then 3 communities on the right panel.

Another network example is the largest component in the eight-letter word subnetwork of the Doublets net. Here we only show its community geometry at two scales as 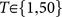 in Fig. 5. The 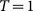 serves as the lowest temperature which yields 8 identified communities, in Fig. 5(a). But only 3 or 4 communities seem solid in connectivity structure, while the remaining ones are mingling with each others. This phenomenon is largely due to the community membership being extracted hierarchical clustering tree with “complete” modular for distance. As 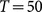, several core communities merge together and yield 3 communities in Fig. 5(b). The mingling effect, seen in Fig. 5(a), is much reduced in this community structure. We believe that the difficulty in computing the community geometry encountered here is primarily caused by the presence of many long dendrites.

### Selection force in the Doublets net

**Figure 6:**
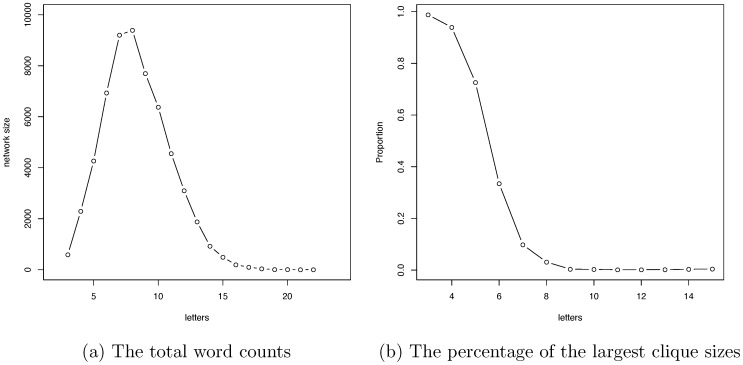
Global information of the Doublets net. The trajectories of word count (a) and percentages of largest clique (b) with respect to letter length exhibit the effects of selection forces on English words as reflected in the Doublets net.

**Figure 7:**
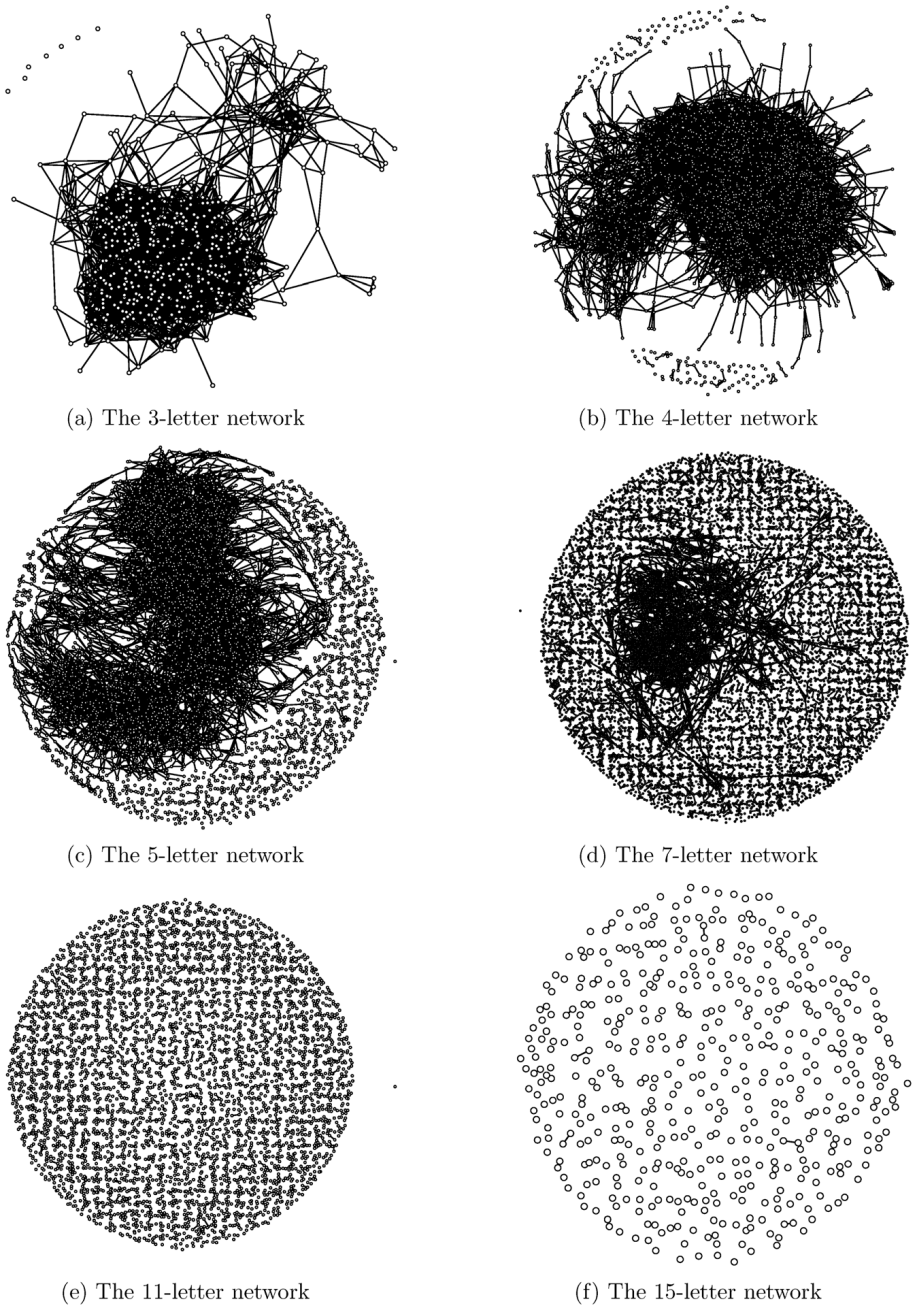
Overview of network dynamics of the Doublets net. Six out of the 20 evolving networks with respect to letter length in the Doublets net are exhibited in six panels. From the nearly complete connectivity in the 3-letter network to having a single large giant clique in 4- and 5-letter networks, to a relatively small clique in the 7-letter network, and then becoming complete scattering in 11- and 15-letter networks.

Finally, we zoom out from individual subnetworks to look at the Doublets net as one whole complex system. Total word counts from 3-letter to 22-letter words are given in Fig. 6(a), while the proportion of the size of the largest clique to the total word count is shown in Fig. 6(b). Among the 20 different letter lengths, we only present six subnetworks to give an overview of the network dynamics of the Doublets net in Fig. 7 (including all singletons). Based on these two figures in Fig. 6, we see that the word count increases with letter length in the 3-letter to 10-letter range. But the word count takes a sharp dive downward after 11-letter words. This up-and-down pattern clearly shows that the language's efficiency goes up as more syllables are used in word structure. But this increasing trend does not last long, as the effect of redundancy appears to overtake the word constructing force after 10-letter words. This is one of the global effects of the selection force on the Doublets net from the perspective of word count.

Another global effect of the selection force is revealed by the ratio of the size of the largest clique to its total word count, as shown in Fig. 6(b) and Fig. 7. The effect drives down the proportion right from 3-letter words onward. It is interesting to note that the decreasing rate peaks with 6-and 7-letter words, which is much earlier than the peak for word count. This pattern shows that, while the efficiency of the English language is optimized through word lengthening, the word-formation schema involving lengthening requires combining existing syllables and morphemes. In sharp contrast, the schema of word formation with 3-and 4-letter words is concerned almost exclusively with the legitimate concatenation of sounds within a single monosyllabic morpheme. Constraints on morpheme and syllable sequencing make long words harder to form than short words. That is why word connectivity is completely lost on very long words, each of which tends to be a singleton in the Doublets net, as shown in Fig. 7. The vivid network heterogeneity that is seen in the structural differences among the strata that are indexed by the different letter lengths of words is reminiscent of the markedly different networking structures that result from the structural sensitivity of hypertext to different web page categories [32].

It should be noted that the maximum size of giant cliques is chosen as the focal index here because the overall network corresponding to each stratum for a given letter-length typically consists of numerous separated and disconnected sub-graphs of widely varying sizes. A small index value means that the English words in the corresponding stratum tend to be isolated, whereas a large index value means that the majority of the words in the corresponding stratum are highly connected. In the latter case, small spelling perturbations of numerous kinds are sufficient to yield potential new words. In the former, much more complex patterns of perturbation are typically required. Because of this, the maximum size of the giant cliques of each stratum-specific network is an informative measure of the systemic network heterogeneity and the underlying selection forces.

It should also be pointed out that this kind of systemic network heterogeneity is distinct from entropy-based network heterogeneity, which has primarily been identified with a single, typically uni-directed, connected network, the definition of which embodies the construct of Shannon's entropy on a probability distribution derived from partitioning networks with respect to graph invariants or vertex functionals (see [33]-[35] for a thorough review and [36] for a proposal concerning a version of network heterogeneity for directed networks).

## Conclusions

Throughout this study the term “selection force” is used as a cover term for phonological, orthographic, and morphological sequencing constraints that increase systemic redundancy for a body of linguistic knowledge about English word structure that is partly manifested in the Doublets net constructed from Lewis Carroll's word game. For this reason the game itself constitutes a complex system in its own right, with multiscale facets at local, intermediate, and global levels that make the underlying selection force computable.

Moreover, the Doublets net is not unusual in terms of its structural and compositional characteristics. In fact, it is similar to many real world complex systems of scientific interest, because the node elements are all different and their relations and edge connections are all distinct. But it distinguishes itself from other complex systems in a crucial way. The knowledge embodied in the Doublets net is a fully “known” system from the perspective of linguistics, whereas most complex systems of interest, if not all, are nearly completely unknown. Thus, the Doublets net is a modular complex system with a potentially invaluable feature, because any computational learning methodology developed for exploring and mining knowledge in an unknown system should first be tested to prove its value and uses in a known modular system. Across a wide range of disciplines, numerous networks have been created to model complex systems in approximation and many claims have been made concerning the promise of network-based solutions to real world problems in the near future. The scientific urgency of such an enterprise is highlighted in a 2009 special section of the journal *Science* (vol. 325, 405–432) that contains 9 articles devoted to connections between complex systems and networks.

Though it is well understood that a complex system under study and its proposed network model need to be shown to be authentically and realistically connected in order to validate claims about the system arising from use of the model, there still seems to exist a big gap between target complex systems and their approximating network models. And, this gap appears not to deter some scientists from expecting to extract useful information from their network models for a wide spectrum of complex systems, ranging from economics to counterterrorism, the biological web of life, the techno-social system and gene transcriptional regulatory circuits.

The problem is that most networks are constructed with mono-type nodes and mono-function edges. This is emphasized in the literature because “agent based models” built upon statistical mechanics of physics are one primary research apparatus used in network analysis. However, the validity of approximating a complex system with a binary network constructed with non-differentiable nodes and edges has not yet been confirmed in any real world case. Consequently, network analysis, in general, is not yet well grounded. At best, any pattern gleaned from such an analysis is typically at the global level and is indiscriminately applied to all nodes. By leaving out the intermediate and local levels of information, the extracted global information could be irrelevant for zoomed-in regions. At worst, much computed information could be extremely misleading due to approximation errors that are not controlled for. The results of community detection techniques based on modularity, as noted in section 1, unfortunately seem to point in the direction of the worst-case scenario. For this reason, information about network structure from a known modular system is of critical importance.

The Doublets net studied here is built upon differentiable nodes and multi-functional edges, which yield both global and local complex system information. Moreover, relevant information is distributed throughout the whole system. Hence it is necessary to do computations by zooming in and out of the Doublets net using various resolutions, in order to deduce the intrinsic multiscale structure of the complex system. This computational learning via multiscale information, as applied to the Doublets net, could be generally required in studies of other complex systems.

When zooming in to a single node in the Doublets net, its local connectivity reveals the relevant constraints on joining and sequencing sounds, syllables, and morphemes in English words. When zooming to an individual subnetwork, its computed geometry of word communities reveals multiscale phonological and orthographic information. When zooming out to view the system as a whole, a balancing act is seen in the constellation of networks indexed by letter length, which evolves from a nearly single giant clique to several less dense cliques, and then transforms into a complete scattering of disconnected single words, and finally an abrupt absence of any words beyond a certain length.

In sum, a good network approximation to a complex system might be best achieved by using differentiable nodes and multi-functional edges. A model of the learning reflected in such systems might also be enriched by orienting computations to multi-scale structural information. And, finally, it is of upmost importance that any proposed computational learning methodology be tested on a known modular system, like the Doublets net. The network and complex system characteristics of the English word-puzzle game studied here may well have seemed obvious and routine to its creator Lewis Carroll. But, the amazing intricacy of its internal structure must be at most barely gleaned by the many people that even today find themselves enchanted by this little wonderland.
